# Influence of Restraint Conditions on Welding Residual Stresses in H-Type Cracking Test Specimens

**DOI:** 10.3390/ma12172700

**Published:** 2019-08-23

**Authors:** Jiamin Sun, Jonas Hensel, Thomas Nitschke-Pagel, Klaus Dilger

**Affiliations:** Institute of Joining and Welding, Braunschweig University of Technology, Langer Kamp 8, 38106 Braunschweig, Germany

**Keywords:** weld cracking, welding processes, welding residual stresses, intensity of restraint, restraint conditions, numerical simulation

## Abstract

From the viewpoint of mechanics, weld cracking tends to occur if the induced tensile stress surpasses a certain value for the particular materials and the welding processes. Welding residual stresses (WRS) can be profoundly affected by the restraint conditions of the welded structures. For estimating the tendency of weld cracking, the small-scale H-type slit joints have been widely used for cracking tests. However, it is still hard to decide whether the real large-scale component can also be welded without cracking even though the tested weld cracking specimens on the laboratory scale can be welded without cracking. In this study, the intensity of restraint which quantitatively indicates how much a joint is restrained is used. The influence of restraint condition (intensity of restraint) on WRS is systematically investigated using both the numerical simulation and the experimental method. The achievement obtained in the current work is very beneficial to design effective H-type self-restrained cracking test specimens for evaluating the sensitivity of the material and the welding procedures for weld cracking in the real large-scale components.

## 1. Introduction

Arc welding is commonly used to join constructional steel in building, offshore structures, and bridges [1]. Residual stress (RS) is unavoidably generated in the weldment because of heterogeneous plastic deformation induced by arc heat input [2,3]. WRS has an evident influence on the initiation and the subsequent growth of cracks [4,5]. To assess the security and integrity of the weldments, it is critical to know the distribution and magnitude of WRS in the designing process.

It is well known that the distribution and magnitude of WRS can be influenced by many factors such as joint types, plate geometry, material properties, welding parameters and restraint conditions [6]. Among those factors, the restraint condition of welded construction has a great influence on WRS [7]. The constraint can either be external from the clamps (fixtures) or be self-induced by the own shape of the welded structures such as the multiple column structures [8]. Thus, understanding the impact of restraint condition on the magnitude and distribution of WRS has become of critical importance in practice.

In recent years, the effect of restraint conditions on WRS has been the focus of attention and has been studied using both the experimental method [9] and the finite element method (FEM) [10,11,12,13]. Teng et al. [14] studied the effect of external mechanical restraint on transverse RS in butt joints. They found that the magnitude of transverse RS in the restrained joint is higher than that in the unconstrained joint. Leggatt [15] summarized the main influential factors on WRS in welded structures. He pointed out that the degree of restraint varies with directions and positions in the weld resulting in the complex distribution of WRS in thick-plate weldments. Liu et al. [16] investigated the influence of the restraint force on WRS. Their work showed that the transverse RS along the weld centerline obviously increases with the constraining force. Heinze et al. [17] numerically and experimentally demonstrated the development of WRS under high restraint conditions in a multi-pass welded joint. They found that in the area next to the weld the transverse RS increases because of the transverse shrinkage restraints, but the transverse shrinkage restraint almost does not affect the longitudinal RS. Hensel et al. [18] analyzed the distribution features of WRS in the constrained and unconstrained weldments and summarized the influential factors on restraint. Farajian [19] compared WRS in the large-scale weldment with the corresponding values in the small-scale test specimen. He showed that just the magnitude of WRS is parallelly shifted in the positive direction for the large-scale sample because of higher structural stiffness in the large-scale specimen resulting in an increased degree of restraint.

Even though many numerical models and experiments have been employed to study the effect of restraint conditions on WRS, the applications of these results from the weldments on the laboratory scale to the analysis of weld cracking in the actual large-scale structures are limited. This is mainly because it is a continuing challenge to confirm the degree of restraint of the real large-scale weldment, which might be restrained by various structural members in practice and would serve under multiple restraint conditions [2,20]. Therefore, the definition of the relationship between the cracking tendency and the degree of restraint at the weld is essential.

For estimating the tendency of weld cracking, a lot of cracking test specimens have been carried out [20]. Nevertheless, it is still hard to confirm whether the actual component can also be welded without cracking even if the small-scale cracking test specimens on the laboratory scale can be joined without cracking [20]. Satoh et al. [2] proposed the intensity of restraint, which quantitatively indicates how much a joint is restrained. The intensity of restraint can determine the degree of restraint in both the cracking test sample (small-scale) and the real welded structure (large-scale), which capacitates the transfer of the restraint condition of the actual welded construction on the simple test weld on the laboratory scale [2,20].

Although there is an unlimited number of factors resulting in weld cracking, the weld crack initiates when the induced tensile stress at a point is higher than the critical value from the mechanical viewpoint [2]. In that case, if the cracking test specimens on the laboratory scale with high WRS can be welded without cracking, it can be expected that the material and the welding parameters applied in the test can also be successfully used in the actual constructions without cracking. Therefore, it is critical to systematically clarify the effect of the intensity of restraint on WRS in the cracking test samples at first.

As fundamental research, a commonly used H-type self-restrained specimen [20,21] was taken as the primary research model. A series of thermal–metallurgical–mechanical numerical analyses were performed to systematically study the influence of restraint conditions on WRS using Simufact.welding 7.0 software [22]. Also, the experiments have been performed to measure thermal cycles and WRS for validation. 

## 2. State of the Art

### 2.1. Self-Restrained Mechanism of the H-Type Slit Joint

**Figure 1:**
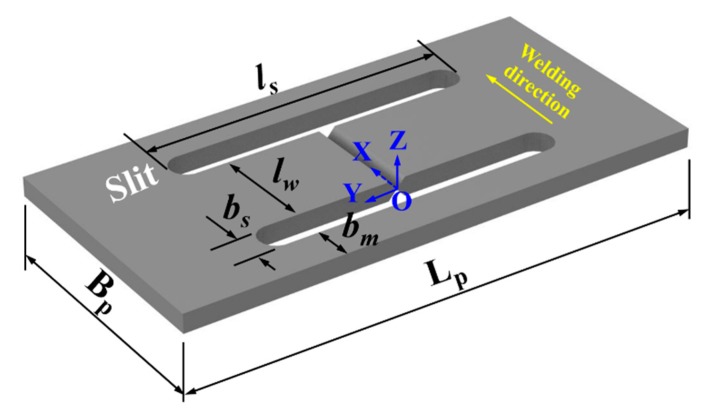
Simple H-type self-restrained specimen.

The model of the simple H-type self-restrained specimen is schematically shown in Figure 1. From Figure 1, it can be found that there are two slits made in the plate perpendicular to the welding direction. Here, the plate geometry L_p_ and *B_p_* are the plate length and width respectively. The dimensions of these two slits are the same, while the slit geometry *l_s_* and *b_s_* represent the slit length and width, respectively. Furthermore, *l_w_* is the weld length and *b_m_* is the plate margin width. It should be noted that the plate thickness and weld seam related factors (welding parameters, welding consumables, groove type, etc.) for the test (small-scale) and actual (large-scale) specimen are the same.

**Figure 2:**
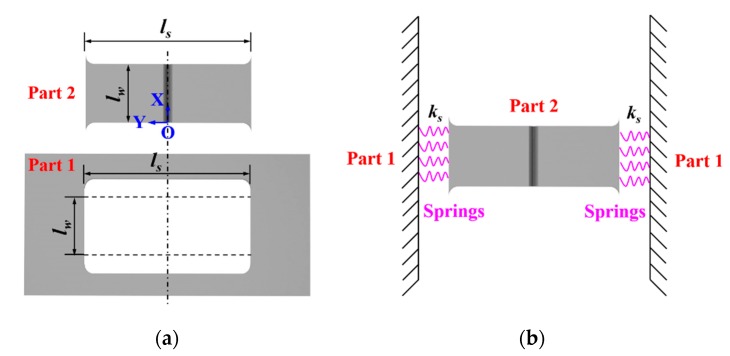
Schematic illustration of the self-restrained mechanism of the H-type slit specimen: (**a**) Combination of the H-type slit joint; (**b**) joint restrained by a set of springs.

The restrained mechanism of the H-type slit joint is schematically illuminated in Figure 2. The H-type specimen can be divided into two parts, Part 1 and Part 2, as in Figure 2a. Part 2 is contained in Part 1, that means the transverse expansion and shrinkage (in *y*-direction) of the weld in Part 2 have been externally restrained by Part 1. It is necessary to mention that the weld in Part 2 has no external restraint in the longitudinal direction (in *x*-direction) here due to the existence of slits as seen in Figure 1. It is well known that the maximum longitudinal residual stress is usually near or equal to the yield strength but the maximum transverse residual stress is rather small [3]. This is mainly because the restraint in the longitudinal direction is much stronger than that in the transverse direction [3]. It would be more meaningful to investigate the influence of the variation in transverse restraint on WRS, especially transverse RS. Therefore, the current H-type model was selected for investigation [21]. Additionally, Part 2 is not entirely rigidly fixed but partially restrained by Part 1 in the transverse direction. The relationship of the restraint between Part 1 and Part 2 can be schematically expressed as a set of springs, as shown in Figure 2b. The spring constant of the restraint is the intensity of restraint (*k_s_*) [20,21] that quantitatively represents the shrinkage constraint of a weld-piece. The *k_s_* of the H-type slit specimen [20,21] is determined by

(1) $$k_{s}=\frac{E}{l_{s}\cdot \left(1+\frac{l_{w}}{2b_{m}}\right)}$$

(2) $$b_{m}=\frac{B_{p}-l_{w}-2\cdot b_{s}}{2}$$

where, *E* is Young’s modulus. Based on Equations (1) and (2), *k_s_* can be expressed as

(3) $$k_{s}=\frac{E}{l_{s}\cdot \left(1+\frac{l_{w}}{B_{p}-l_{w}-2\cdot b_{s}}\right)}$$

From Equation (3), one can see that *k_s_* is mainly determined by E and geometric parameters. The geometric parameters here are *l_w_*, *l_s_*, *b_s_* and *B_p_*, as seen in Figure 1.

As *E* (Young’s modulus) can be regarded as a known parameter if the material is determined, *k_s_* is just varied with geometric parameters. Furthermore, the purpose of the slits made here is to let the weld in the longitudinal direction be free from the external restraint, and the value of *b_s_* is relatively small. Also, the influence of *b_s_* on WRS can be reflected by that of *l_w_*. Thus, *b_s_* can also be treated as a known value here. Through the careful observation in Figure 1, one can see that the variation range of *l_w_* is related to *B_p_*. Similarly, that of *l_s_* is related to L_p_. Thus, if the plate geometry (L_p_, *B_p_*) is reasonable and is fixed, the influence of the degree of restraint (intensity of restraint, *k_s_*) on WRS can be fully displayed just by the changes of *l_w_* and *l_s_*. Based on the above analysis, the restraint condition (intensity of restraint, *k_s_*) is changed only by the variation in *l_w_* or *l_s_* in the present study.

### 2.2. Effect of Restraint Direction on Residual Stresses

**Figure 3:**
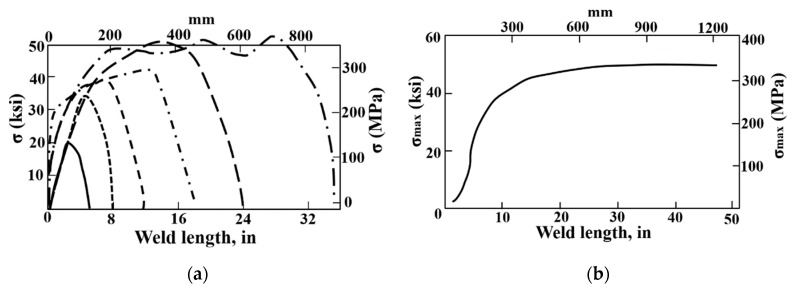
Effect of weld length on longitudinal residual stress (from [23]): (**a**) Longitudinal residual stress distributions; (**b**) maximum longitudinal residual stress.

As the variation in restraint condition here is in the transverse direction, as seen in Figure 2, it can be expected that the magnitude of transverse RS can be affected directly by the change of *k_s_* here. Through the investigation on the impact of the weld length on WRS by DeGarmo et al. [23], one can learn that the longitudinal residual stress can reach its extreme value (yield strength) if the weld length is longer than the critical weld length [23], as seen in Figure 3. The critical weld length increases in proportion to the width of the plastic strain zone [24], which is also directly proportional to *k_s_*. Therefore, the maximum longitudinal residual stress could be indirectly affected, even though the restraint condition changes in the transverse direction here.

## 3. Materials and Methods

**Figure 4:**
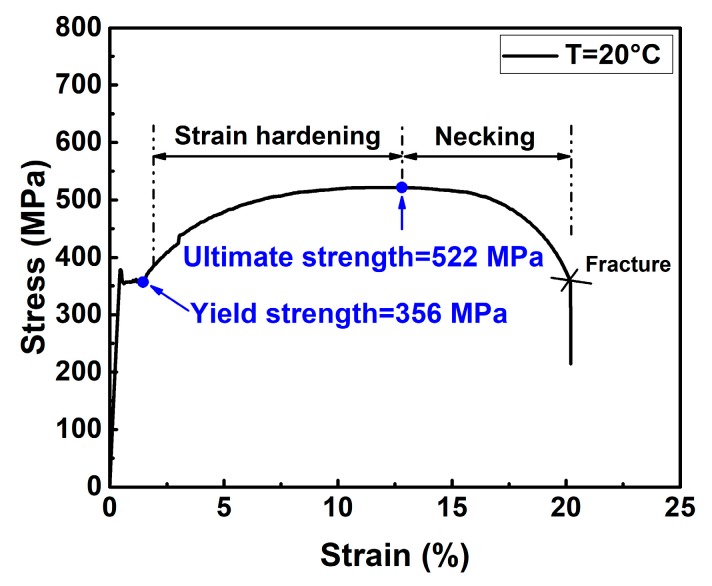
The measured stress-strain curve of the base metal.

**Figure 5:**
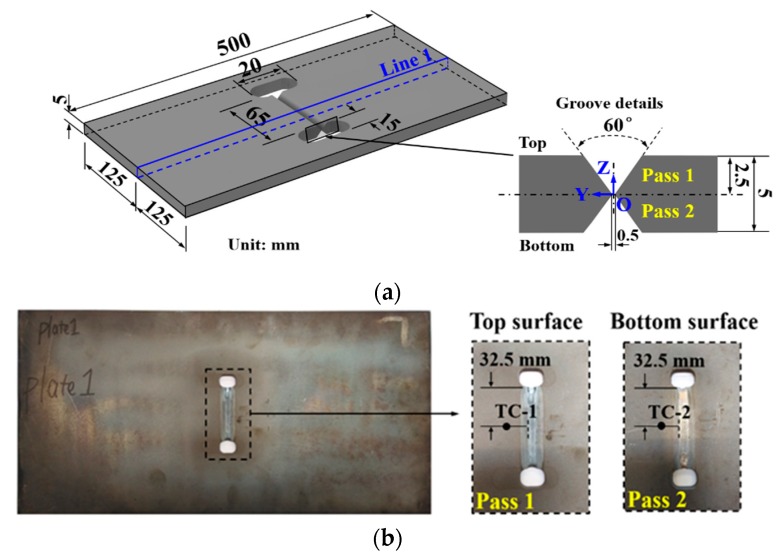
The dimension of the experimental mock-up: (**a**) Schematic diagram; (**b**) experimental specimen.

**Figure 6:**
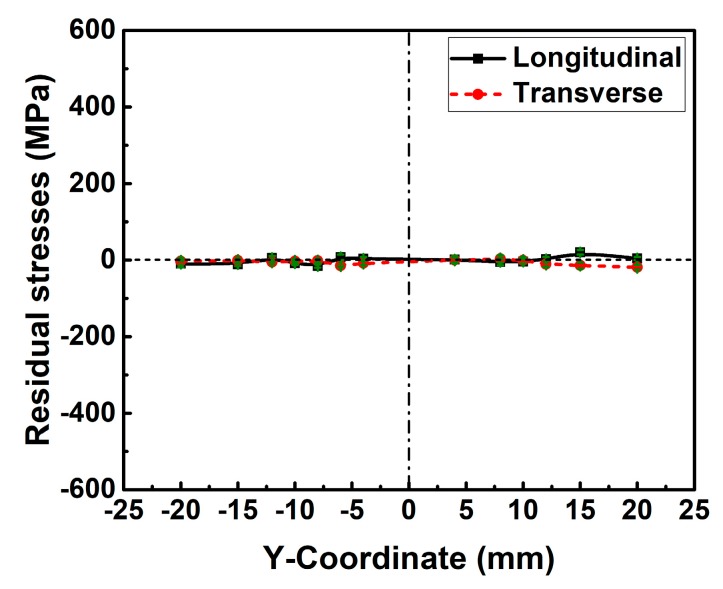
The measured distribution of longitudinal and transverse residual stress along line 1 after heat treatment.

The base metal is the commonly used structural steel S355N, and the filler material is G4Si1 (EN 440 standard). The measured chemical composition of S355N steel is shown in Table 1. The measured stress-strain curve for this material from the tensile test is shown in Figure 4. The measured yield strength of the base metal (ferrite–pearlite phase) is about 356 MPa. The designed plate geometry here is shown in Figure 5, and that is 500 mm × 250 mm × 5 mm. Before welding, the plate was heated to about 600 °C at about 5 °C/min for one hour followed by furnace cooling to room temperature (about 20 °C) under an argon atmosphere for eliminating the initial stress caused by the manufacturing process. After the heat treatment, both longitudinal and transverse RS distributions along line 1 (see Figure 5) were measured by the X-ray diffraction method and the experimental results are shown in Figure 6. From Figure 6, one can see that both longitudinal and transverse RS were close to 0 MPa. The specimen was welded by gas tungsten arc welding (GTAW) performed by a DALEX VARIO TIG 400 robot. Figure 5 describes the deposition sequences, and the welding directions of pass 1 and pass 2 are the same. Table 2 provides the applied welding parameters. The inter-pass temperature was lower than 150 °C. Note that the mock-up was welded without any external fixtures. 

K-type thermocouples were used to measure the thermal cycles, and the locations of thermocouples are reported in Figure 5b and Table 3. Here, TC is the abbreviation of the thermocouple. After welding, WRS along line 1 on the top surface of the mid-cross section (see Figure 5) was measured by the X-ray diffraction method.

## 4. Finite Element Analysis

**Figure 7:**
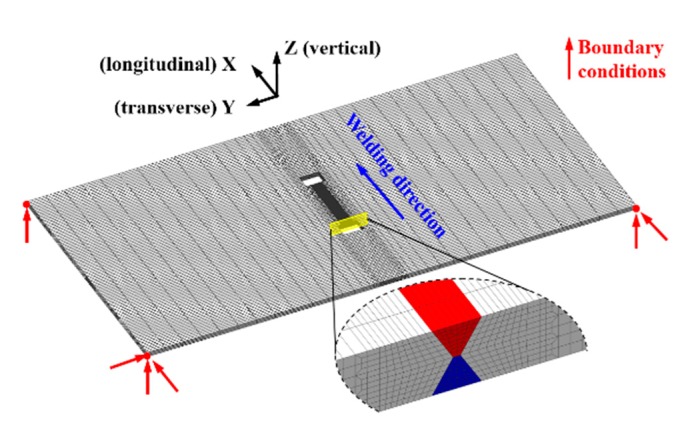
Finite element mesh and mechanical boundary conditions.

Figure 7 shows the finite element model. The size of this model is the same as that of the experimental specimen. For balancing the simulation accuracy and calculation time, the finer mesh was designed only in and near the weld, as seen in Figure 7. The finite element model was meshed consists of 26,496 elements and 33,926 nodes. The smallest mesh is about 0.5 mm × 0.5 mm × 2.5 mm. The element type applied in the numerical analysis is type 7 (eight-node cubic element) [22].

### 4.1. Thermal Analysis 

**Figure 8:**
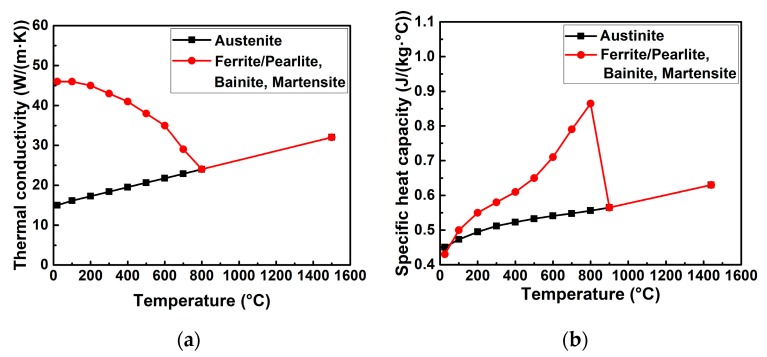
Temperature-dependent thermal properties of S355 steel (from [22]): (**a**) Thermal conductivity; (**b**) specific heat capacity.

The classic Goldak’s double-ellipsoid volumetric heat source was applied to compute the welding temperature field [10]. The used thermal properties were taken from the Simufact.welding material database as shown in Figure 8 [22]. It is necessary to mention that the base material and the filler wire were assumed to have the same material properties. The latent heat was considered in the simulation, while the value of latent heat was 256.4 J/g [22]. The heat losses due to convection and radiation were also taken into account [22]. The convective heat transfer coefficient (h) was 20 W/(m^2^·K), and the emission coefficient (ε) was 0.6 [22]. Here, the room temperature was about 20 °C, the metallurgical melting temperature was assumed as 1500 °C, and the peak temperature arrived in the heat-affected zone (HAZ) was from 750 to 1500 °C [3,25].

### 4.2. Mechanical Analysis 

**Figure 9:**
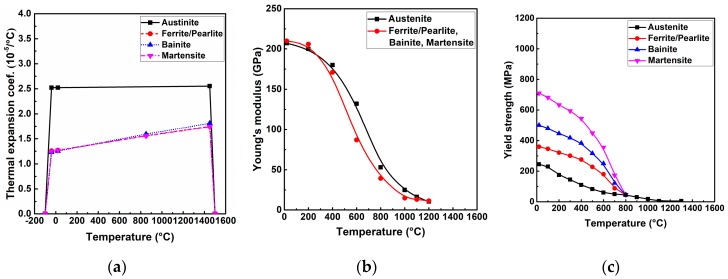
Temperature-dependent mechanical properties of S355 steel (from [22]): (**a**) Thermal expansion coefficient; (**b**) Young’s modulus; (**c**) yield strength.

The mechanical boundary condition is shown in Figure 7 for preventing the rigid body motion. In the present study, the solid-state phase transformation (SSPT) was considered for S355 steel [22,25]. The diffusive transformation was modeled by the Johnson–Mehl–Avrami–Kolmogorov (JMAK) kinematic equation [26,27,28]. Because of the used JMAK model, the time–temperature–transformation (TTT) diagram was used combined with the application of the notion of fictitious time and the scheil’s additivity principle to track the phase evolution during non-isothermal diffusive transformation. The displacive transformation was imitated by the Koistinen–Marburger (KM) relationship equation [29]. The used mechanical properties were taken from the Simufact.welding material database, as shown in Figure 9 [22]. Note that the material properties of the generated phase mixture were determined by the linear mixture rule. The strain hardening was ignored in the simulations here [25].

### 4.3. Simulated Cases 

As analyzed in Section 2.1, the variation in the restraint condition is caused by the change in *l_s_* and *l_w_* in this study. The designed simulation cases here are shown in Table 4. In Table 4, these cases are divided into four groups. In each group, *l_s_* varies while *l_w_* is kept as constant. Furthermore, those cases in Table 4 can also be regrouped. The cases A-1, B-1, C-1, and D-1 can be collected into a new group named group 1. Then, *l_s_* of those cases in group 1 is 20 mm, while *l_w_* varies from 45 to 105 mm. Similarly, groups 2–5 can also be created.

## 5. Comparison between Experimental and Simulated Results

### 5.1. Welding Temperature Field

**Figure 10:**
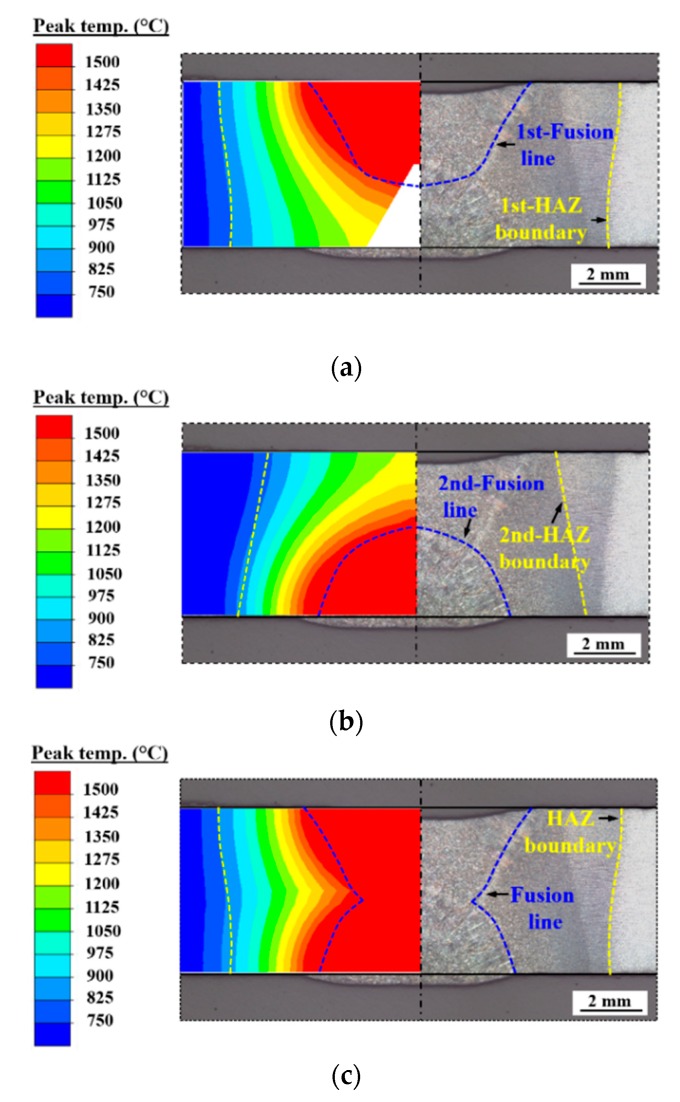
Comparison of the weld between experiment and FEM (case B-1): (**a**) Pass 1; (**b**) pass 2; (**c**) all passes.

**Figure 11:**
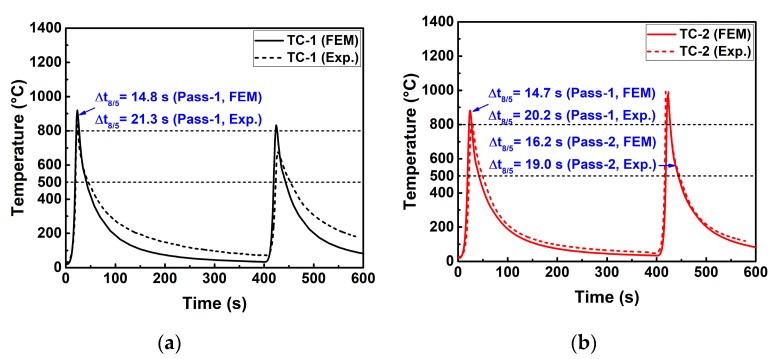
Comparison of the thermal cycle between experiment and FEM (case B-1): (**a**) At TC-1 location; (**b**) at TC-2 location.

Figure 10 compares the weld dimensions in the mid-cross section predicted by case B-1 with the experimental results. In Figure 10, both the shape and size of FZ and HAZ computed by case B-1 are in good agreement with the measurements. Figure 11 compares the simulated and measured temperature histories at TC-1 and TC-2 locations (see Table 3). In Figure 11, the thermal cycles show a satisfactory agreement between the predictions and the measurements. Table 5 compares the calculated phase fractions with the empirical values which are from the predictive software Weldware using the measured chemical composition (see Table 1) and cooling rate Δt_8/5_ time (see Figure 11). In Table 5, the predictions match the empirical values very well. It is well known that the chemical compositions among the same steel grades are often different, which can affect the thermal properties [30]. Since it is time-consuming and costly to directly measure material properties for a given material, the most commonly used method in numerical simulation is to take the measurements of the same steel grades from published scientific papers usually included in commercial finite element software material databases. This might be the leading cause for the slight deviation between the predicted and measured cooling rate Δt_8/5_ time. Nevertheless, no significant microstructural differences are obtained for this deviation of cooling rate Δt_8/5_ time. Thus, it can be summarized that the FEM used here can reasonably reproduce the welding temperature field.

### 5.2. Welding Residual Stresses

**Figure 12:**
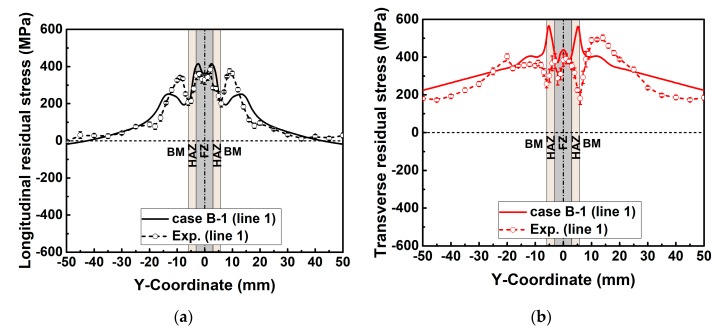
Welding residual stress distributions along line 1: (**a**) Longitudinal residual stress; (**b**) transverse residual stress.

Figure 12 contrasts the simulated and measured welding residual stress distributions along line 1 (see Figure 5a). In Figure 12, the calculated longitudinal and transverse residual stress match the measurements well both in the magnitude and in the distribution overall. Therefore, the used numerical method here is reliable.

## 6. Influence of Restraint Conditions on Welding Residual Stresses

**Figure 13:**
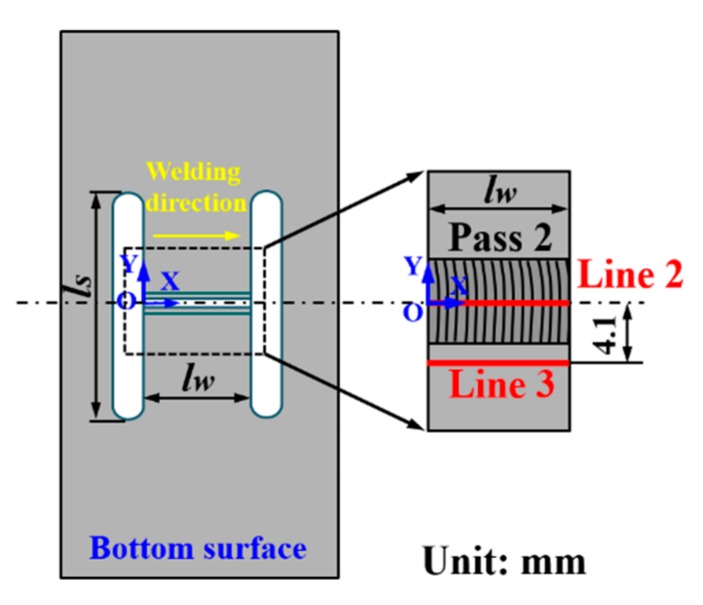
Locations of line 2 and line 3.

It is stressed again that the restraint condition (intensity of restraint, *k_s_*) is changed only by the variation in *l_s_* and *l_w_* in the present study (refer Section 2.1 for detail). Since the cold cracks mainly occur within the FZ or in the HAZ [20], the welding residual stress distributions along line 2 in the FZ and line 3 in the HAZ (as shown in Figure 13) are extracted for analysis. 

### 6.1. Slit Length (l_s_)

**Figure 14:**
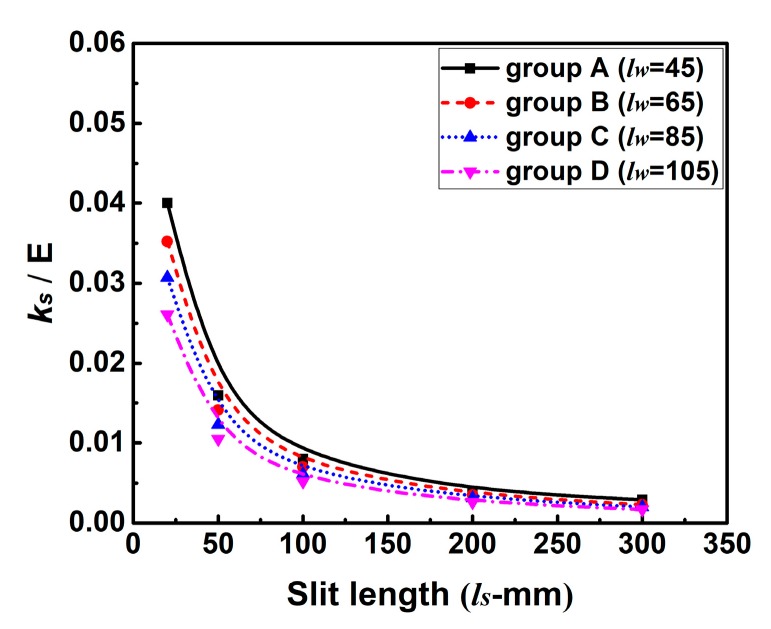
Influence of slit length (*l_s_*) on the intensity of restraint (*k_s_*).

Here, the influence of the variation in *k_s_* induced by *l_s_* on WRS has been studied numerically. According to Equation (3) and Table 4, the theoretical effect of *l_s_* on *k_s_* can be found in Figure 14. In this figure, *k_s_* in each group decreases with *l_s_* sharply at first but then drops down slowly. Note that the change of the restraint condition here is in the transverse direction.

**Figure 15:**
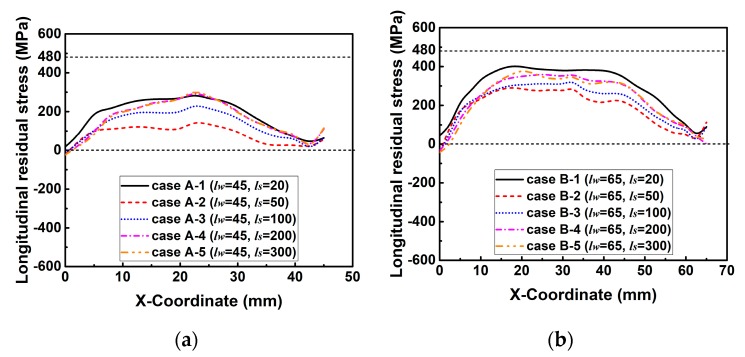

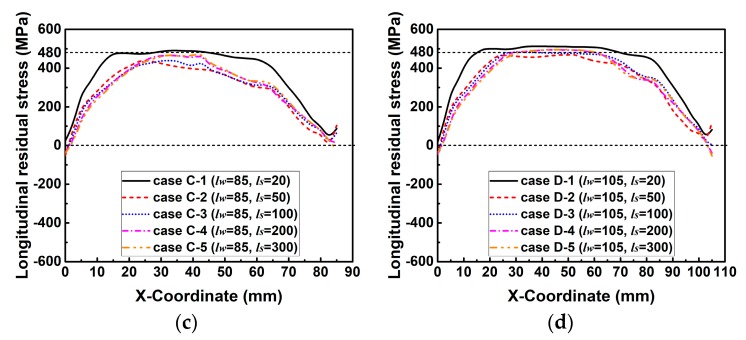
Longitudinal residual stress distributions along line 2 in the cases of each group with varied slit length (*l_s_*) while the weld length (*l_w_*) is kept constant: (**a**) Group A; (**b**) group B; (**c**) group C; (**d**) group D.

Figure 15 compares the predicted longitudinal RS distributions along line 2. The yield limit of the weld is about 480 MPa based on the linear mixture rule. From Figure 15, one can see that the magnitudes of longitudinal RS in those cases of group A and group B are all lower than the yield strength (480 MPa), while those of group C and group D reach the maximum value. This is because the longitudinal RS can reach its extreme value only if the weld length is longer than the critical weld length, which is directly proportional to *k_s_* (refer to Section 2.2 for details). From Figure 15, one can deduce that the critical weld length varied by *k_s_* here would be higher than 65 mm but less than 85 mm in this study.

**Figure 16:**
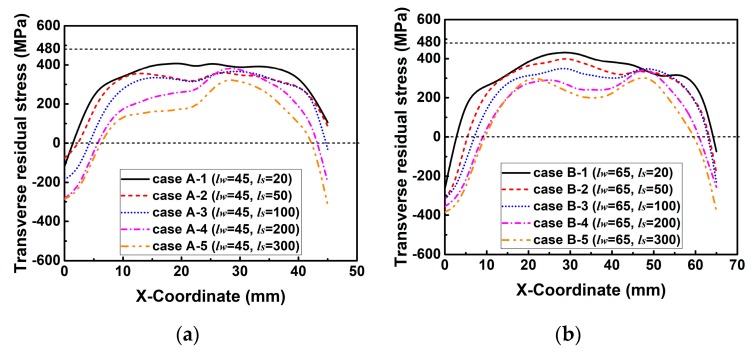

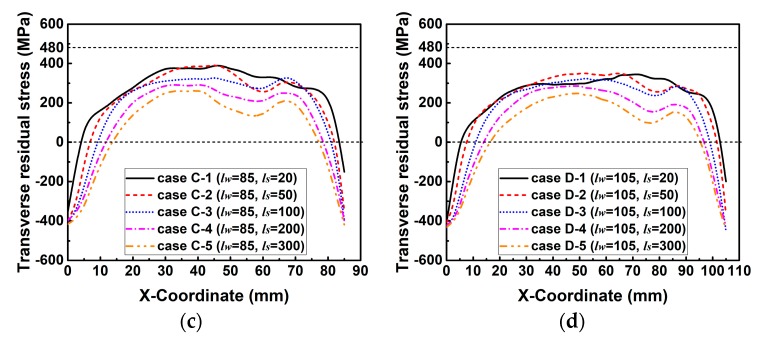
Transverse residual stress distributions along line 2 in the cases of each group with varied slit length (*l_s_*) while the weld length (*l_w_*) is kept constant: (**a**) Group A; (**b**) group B; (**c**) group C; (**d**) group D.

**Figure 17:**
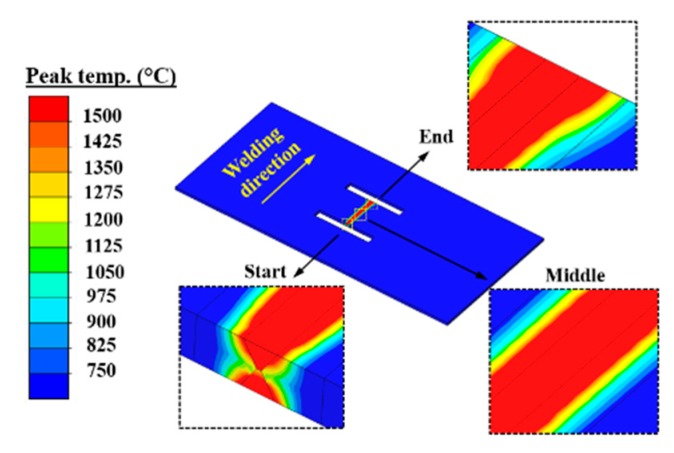
The contour of peak temperature distribution in case B-3.

Figure 16 describes the simulated transverse RS distributions along line 2. In Figure 16, the magnitude of transverse RS decreases with the increase of *l_s_* in each group. This is because *k_s_* decreases with the increase of *l_s_*, as shown in Figure 14. The decreasing *k_s_* can increase the degree of freedom in deformation, and thereby reduces the transverse RS [3,31]. Through careful observation, the transverse RS distribution shows a wave at the end of the weld, especially in the case of a lower *k_s_* or a longer *l_s_*. This is because the width of higher temperature area at the weld end is wider than that at the start and in the middle of the weld as shown in Figure 17, which is due to the reduction of heat conduction area at the weld end. Since the shrinkage force is directly proportional to the welding temperature, the transverse shrinkage increases along the welding direction [24]. The transverse shrinkage at the weld end then is greater than that at other places if the weldment is allowed to deform freely. However, the transverse shrinkage of Part 2 was externally constrained by Part 1 here, as shown in Figure 2. In that case, a higher restrained plastic strain can be obtained at the weld end causing the higher transverse RS [3,31]. Therefore, transverse RS distribution forms a wave shape at the weld end here.

**Figure 18:**
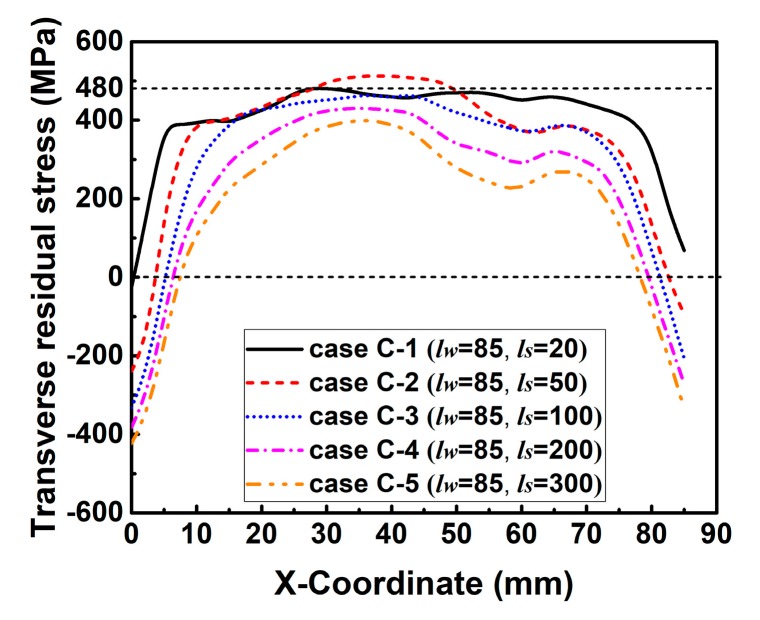
Transverse residual stress distribution along line 3 in cases of group C.

Since the distribution regulations of transverse RS in those groups are similar, only the transverse RS distribution along line 3 in the HAZ (see Figure 13) predicted by those cases in group C are plotted in Figure 18. From Figure 18, it can be found that the magnitude of transverse RS also decreases with the increase of *l_s_* overall. Furthermore, the maximum transverse RS in these cases reach or are near the yield limit. 

### 6.2. Weld Length (l_w_)

**Figure 19:**
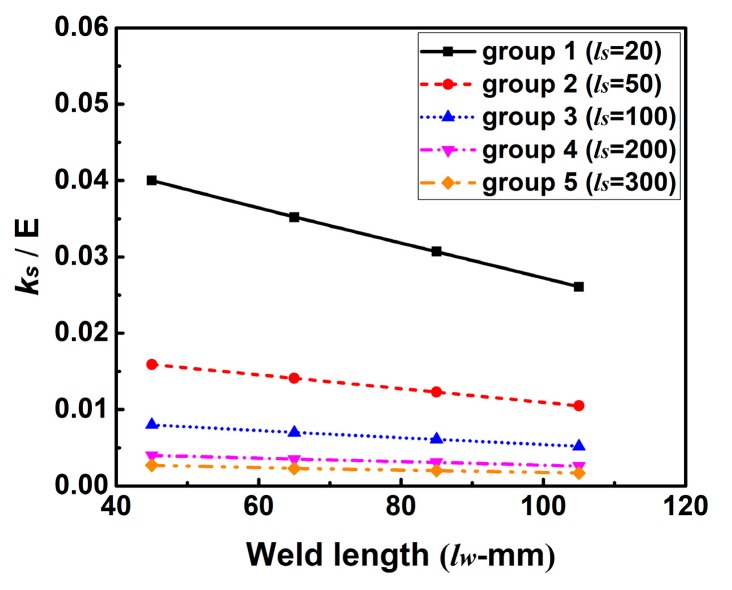
Influence of weld length (*l_w_*) on the intensity of restraint (*k_s_*).

In this section, the impact of the change of *k_s_* caused by *l_w_* on WRS has been investigated using FEM. The theoretical effect of *l_w_* on *k_s_* is plotted in Figure 19. From Figure 19, one can see that *k_s_* decrease linearly with the increase of *l_w_*. In addition, it is obvious that the descent rate of *k_s_* decreases with the increase of *l_s_*.

**Figure 20:**
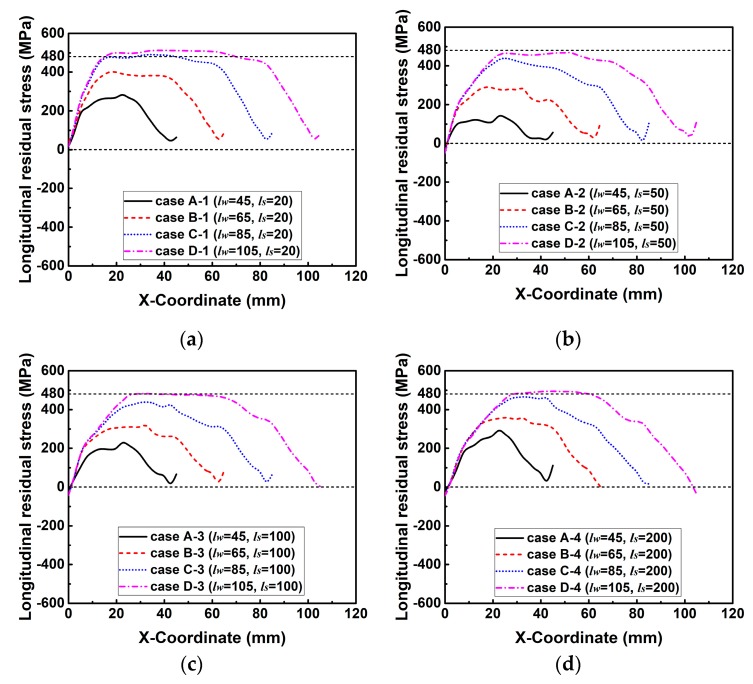

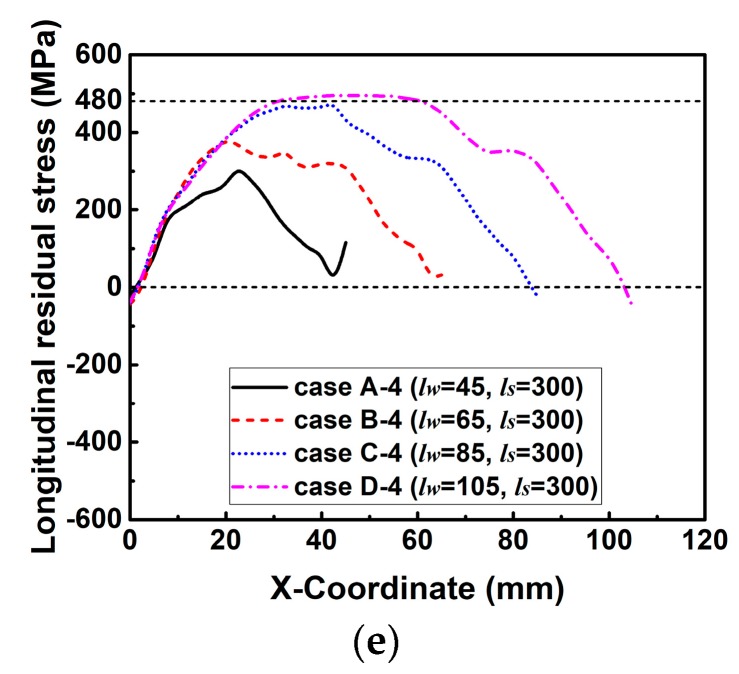
Longitudinal residual stress distributions along line 2 in the cases of each group with varied weld length (*l_w_*) while the slit length (*l_s_*) is kept constant: (**a**) Group 1; (**b**) group 2; (**c**) group 3; (**d**) group 4; (**e**) group 5.

Figure 20 illuminates the predicted longitudinal RS distributions along line 2. From Figure 20, it can be found that the change tendency of longitudinal RS distribution caused by the variation in the degree of restraint in these groups is comparable. Here, Figure 20a is taken as an example to describe the distribution mechanism of longitudinal RS. From Figure 20a, one can see that the magnitude of longitudinal RS in case A-1 is the lowest, that in case B-1 increases but is still smaller than the maximum value (480 MPa), while that in case C-1 and case D-1 reach the yield strength. This is because the weld lengths in case A-1 and case B-1 are all smaller than the critical weld length, while those in case C-1 and case D-1 are longer than the critical weld length (refer to Section 2.2 for details).

**Figure 21:**
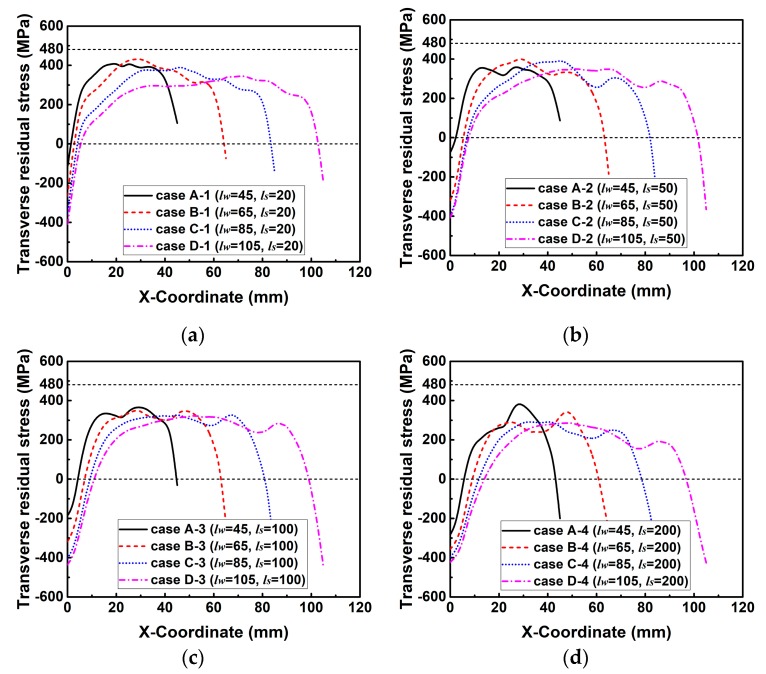

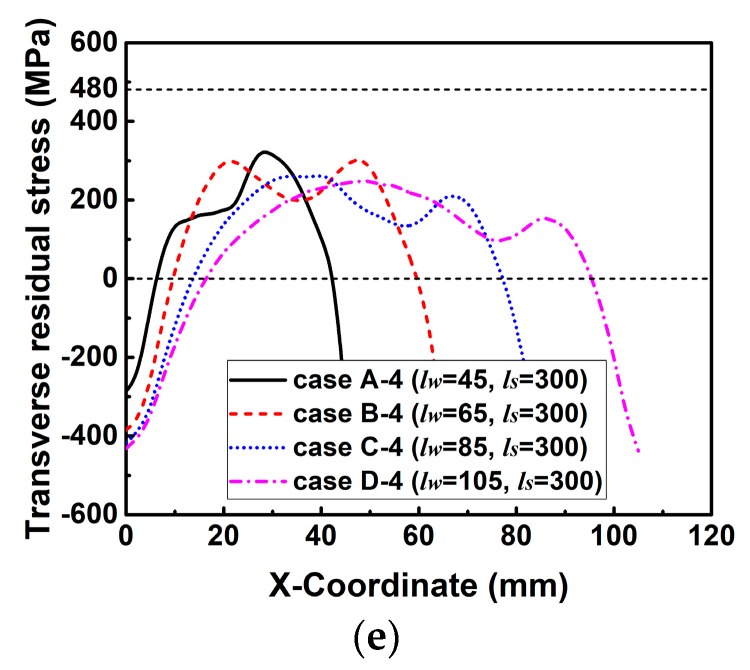
Transverse residual stress distributions along line 2 in the cases of each group with varied weld length (*l_w_*) while the slit length (*l_s_*) is kept constant: (**a**) Group 1; (**b**) group 2; (**c**) group 3; (**d**) group 4; (**e**) group 5.

Figure 21 shows the simulated transverse RS distributions along line 2. From Figure 21, it can be seen that the magnitude of transverse RS in those cases of each group decreases with the increase of *l_w_*. This is because the *k_s_* decreases with increasing *l_w_* as shown in Figure 19. Through the careful comparison, one can see that the maximum difference of the magnitude of transverse RS in each group decreases from group 1 to group 5. This is due to the different descent rate of *k_s_* in these groups as seen in Figure 19.

**Figure 22:**
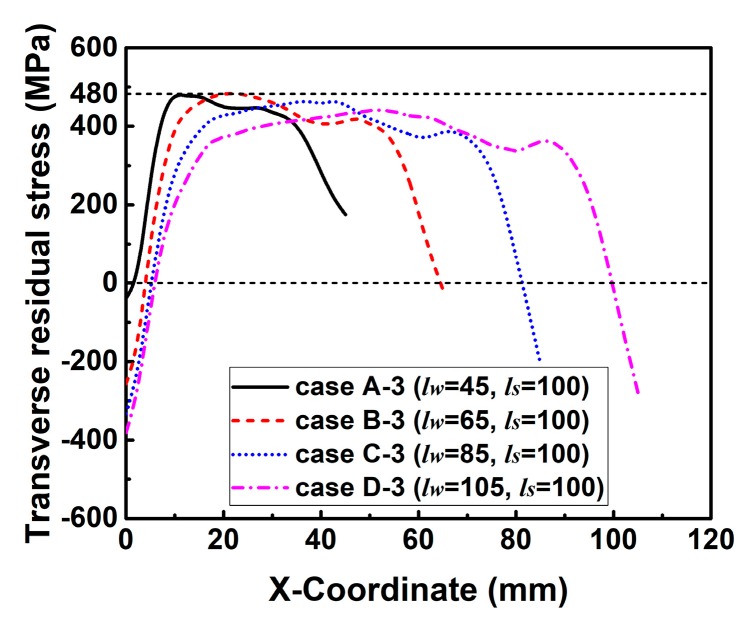
Transverse residual stress distribution along line 3 in cases of group 3.

Figure 22 describes the transverse RS distributions along line 3 predicted by those cases in group C. In Figure 22, the magnitudes of transverse RS along line 3 in those cases reach or are close to the yield strength, and the distributions of transverse RS are almost the same. 

## 7. Discussion

From the mechanical viewpoint, the weld crack initiates if the produced tensile stress/strain at a point reaches the critical value [2]. Based on the current developed approach, to assess the sensitivity of the material and welding parameters for weld crack, the best strategy is to find out the cases with higher magnitude of welding (tensile) residual stresses in the restrained test specimens by FEM, and then to perform these weld cracking experimental tests. If the restrained joints with the higher tensile residual stress can be welded without weld cracking, it can be expected that the used material and the welding procedure in the test can also be successfully applied in the actual constructions. 

According to the current study, one can see that the restraint condition in the small-scale H-type self-restrained joint could easily vary in an extensive range by the change of H-type sample geometry. The variation in the restraint condition has a noticeable effect on the magnitude of transverse residual stress, which is directly proportional to the intensity of restraint until that reaches the extreme value (yield strength). Furthermore, the change of restraint condition in the transverse direction can also influence the magnitude of longitudinal residual stress, but this influence can appear only in the model with a shorter weld length that is less than the critical weld length.

Based on the above results analysis, it was found that the longitudinal RS can reach the maximum value if the weld length is longer than 85 mm here. The magnitude of transverse residual stress decreases if the slit length increases or the weld length increases usually. Therefore, case C-1 with 85 mm weld length and 20 mm slit length had the highest both longitudinal and transverse residual stress in the weld here. In that case, if case C-1 can be welded without cracking in this study, one can deduce that the currently applied material and the welding parameters can also be used in the real construction.

For a lightweight design, the high-strength structural steels such as S690 and S960 steel have become more and more widely applied in actual construction. However, these high-grade steels tend to be more sensitive to the weld cracking than the mild steels [32]. Therefore, further investigation on the influence of restraint conditions on WRS in the high-strength steels will be carried out in the near future.

## 8. Conclusions

(1) The magnitude of longitudinal residual stress can be affected by the variation in the intensity of restraint (degree of restraint), but this influence can appear only in the model with the shorter weld length.

(2) The restraint condition has a significant impact on the magnitude of transverse residual stress. And, the magnitude of transverse residual stress is directly proportional to the intensity of restraint until that reaches the extreme value (yield strength).

(3) Through this study, it becomes possible to design small-scale laboratory test specimens reflecting the influence of restraint conditions on welding residual stresses in the real large-scale welded components.

(4) The achievement obtained in this study is very useful to design effective H-type restrained cracking tests for assessing the sensitivity of the particular material and the welding procedures for weld cracking.

## Figures and Tables

**Table 1:** Measured chemical composition of S355N steel.

Elements	C	Si	Mn	P	S	Cu
Value	0.121	0.315	1.42	0.0108	0.0039	0.0449
Elements	Mo	Ni	Al	Ti	Cr	Fe
Value	0.0017	0.0204	0.0301	0.0011	0.0338	Balance

**Table 2:** Welding parameters.

Pass	Current (I/A)	Voltage (U/V)	Welding Speed (mm/s)	Wire Feeding Speed (mm/s)
Pass 1	132	13.0	1.67	9.25
Pass 2	132	13.0	1.67	9.25

**Table 3:** Locations of K-type thermocouples.

Number	Distance from Fusion Line (mm)
TC-1 (Top surface)	2.42
TC-2 (Bottom surface)	1.95

**Table 4:** Simulation cases with varied weld length (*l_w_*) and slit length (*l_s_*).

Cases		*l_w_* (Weld Length)	*l_s_* (Slit Length)
group A	case A-1	45	20
	case A-2		50
	case A-3		100
	case A-4		200
	case A-5		300
group B	case B-1	65	20
	case B-2		50
	case B-3		100
	case B-4		200
	case B-5		300
group C	case C-1	85	20
	case C-2		50
	case C-3		100
	case C-4		200
	case C-5		300
group D	case D-1	105	20
	case D-2		50
	case D-3		100
	case D-4		200
	case D-5		300

**Table 5:** Phase percentages referring to a node at the middle of FZ.

Phase Fraction (wt.%)	Ferrite-Pearlite	Bainite	Martensite
Case B-1	12	88	0
Empirical value	21	79	0
